# Perfusion Index and Hemodynamic Responses During Propofol Induction with Fentanyl or Ketamine–Lidocaine Adjuncts: A Prospective Randomized Pilot Clinical Study

**DOI:** 10.3390/medicina62081443

**Published:** 2026-07-25

**Authors:** Mara Klibus, Rajesh Prabhakar Bhavsar, Jekaterina Jagodzinska Peskova, Niklavs Nemme, Jelena Duboka, Liga Gabrane, Olegs Sabelnikovs

**Affiliations:** 1 Department of Anaesthesiology, Intensive Care and Clinical Simulations, Riga Stradiņš University, 1007 Riga, Latviaolegs.sabelnikovs@rsu.lv (O.S.); 2 Department of Anaesthesiology, Pauls Stradins Clinical University Hospital, 1002 Riga, Latvia; 3 Department of Anesthesia and Critical Care Medicine, South Jutland University Hospitals, 6200 Aabenraa, Denmark; rajesh.prabhakar.bhavsar@rsyd.dk (R.P.B.); jekaterinajagodzinska@gmail.com (J.J.P.)

**Keywords:** perfusion index, induction of anesthesia, hemodynamic stability

## Abstract

*Background and Objectives:* Hemodynamic instability during anesthesia induction and endotracheal intubation remains a clinically important concern. Low-dose ketamine and intravenous lidocaine may provide complementary effects that improve cardiovascular stability and peripheral perfusion. This pilot study evaluated effects of adding ketamine and lidocaine to a propofol-based induction regimen using mean arterial pressure (MAP) and perfusion index (PI) as monitoring parameters. *Materials and Methods:* In this prospective observational pilot study, 30 adult patients undergoing elective surgery requiring general anesthesia and endotracheal intubation were allocated to either a standard induction regimen (Group 1: propofol 2 mg/kg and fentanyl 2 μg/kg) or an adjunctive regimen (Group 2: propofol 2 mg/kg, ketamine 0.4 mg/kg, lidocaine 1 mg/kg, and fentanyl 1 μg/kg). Hemodynamic variables and PI were recorded at baseline, after induction, and after intubation. Postoperative sore throat and cough-reflex responses were also assessed. *Results:* No significant differences between-groups were observed in heart rate, systolic blood pressure, diastolic blood pressure, MAP, or oxygen saturation at any time point. However, MAP decreased significantly within Group 1 after induction (94.9 ± 10.1 vs. 76.9 ± 13.8 mmHg, *p* < 0.001) and remained lower after intubation (*p* = 0.001), whereas no significant MAP changes occurred within Group 2. PI increased in both groups but was significantly higher in Group 2 after induction (5.35 ± 2.34 vs. 3.46 ± 2.90, *p* = 0.043) and after intubation (7.02 ± 2.89 vs. 4.71 ± 2.22, *p* = 0.020). Postoperative sore throat scores were lower in Group 2 (0.53 ± 0.91 vs. 1.67 ± 1.76, *p* = 0.035), and cough-reflex scores were also reduced (*p* = 0.024). *Conclusions:* The addition of low-dose ketamine and intravenous lidocaine to propofol-based induction was associated with higher perfusion index values and reduced airway-related adverse effects. No statistically significant between-group difference in mean arterial pressure (MAP) was detected. These findings are hypothesis-generating and warrant confirmation in larger randomized trials.

## 1. Introduction

Over recent decades, anesthesia-related complications and mortality have declined substantially, despite an increasing number of older and higher-risk patients undergoing surgery [1]. Okolo et al. reported that anesthesia-attributable mortality decreased from approximately 357 per 1,000,000 anesthetics before the 1970s to approximately 34 per 1,000,000 anesthetics in more recent periods [2]. This improvement reflects major advances in anesthetic practice, monitoring, pharmacology, airway management, and perioperative safety systems [3]. However, anesthetic induction and endotracheal intubation still remain physiologically vulnerable periods, during which rapid changes in anesthetic depth, autonomic tone, vascular resistance, myocardial performance, and airway stimulation may produce clinically relevant hemodynamic responses [4].

Propofol (2,6-diisopropylphenol) is one of the most commonly used intravenous agents for induction of anesthesia because of its rapid and reliable onset, short duration of action, and favorable recovery profile [5]. However, propofol administration is frequently associated with hypotension, systemic vasodilation, and myocardial depression [6,7]. Conversely, laryngoscopy and endotracheal intubation may provoke an acute sympathetic response, resulting in hypertension, tachycardia, coughing, and increased perioperative stress [8]. These opposing physiological effects create a clinical challenge: achieving sufficient anesthetic depth to suppress airway reflexes and intubation responses while maintaining cardiovascular stability. This balance is particularly relevant in older patients and in those with cardiovascular disease, impaired autonomic regulation, or limited physiological reserve, in whom even short periods of hemodynamic instability may be undesirable [8].

Propofol combined with an opioid such as fentanyl remains a widely used and clinically rational induction strategy. Fentanyl reduces propofol dose requirements, improves suppression of airway reflexes, and attenuates the sympathetic response to laryngoscopy and endotracheal intubation [9]. However, these beneficial effects are accompanied by sympatholytic and vagotonic actions, which may contribute to bradycardia, hypotension, or exaggerated cardiovascular depression, particularly when combined with propofol or used in patients with limited physiological reserve [10,11]. In routine clinical practice, such instability is often managed with fluids, phenylephrine, ephedrine, or other vasoactive interventions. However, these interventions are primarily reactive i.e., they correct hypotension or circulatory disturbance after it has occurred, rather than preventing or attenuating the initial pharmacological and autonomic perturbation. Therefore, the clinical question is not whether propofol–fentanyl induction is appropriate, but whether complementary adjuncts can provide a more balanced induction profile by reducing the magnitude of early hemodynamic disturbance and potentially limiting the need for corrective vasoactive treatment.

In this context, low-dose ketamine and intravenous lidocaine represent potentially useful adjuncts because their pharmacological profiles may complement the limitations of propofol–opioid induction. Ketamine has predominant sympathomimetic properties and may support arterial pressure and heart rate through central nervous system stimulation, while providing anesthesia, sedation, and analgesia with relative preservation of cardiopulmonary stability and airway patency [12]. However, ketamine alone may not reliably suppress airway reflexes or the pressor response to intubation [13]. Intravenous lidocaine may provide a complementary effect by attenuating airway reflexes, coughing, and the hemodynamic response to laryngoscopy and endotracheal intubation [14,15,16,17]. At therapeutic intravenous concentrations, lidocaine also blocks voltage-gated sodium channels and modulates muscarinic and NMDA receptor activity, contributing to analgesic, anti-hyperalgesic, and antinociceptive effects beyond local anesthesia [18]. Furthermore, previous studies suggest that lidocaine-based induction may reduce postinduction hypotension in older patients compared with fentanyl, and that a lidocaine–ketamine combination may reduce hypotension compared with full-dose ketamine during rapid-sequence intubation in septic shock [19,20]. Together, these properties suggest that a ketamine–lidocaine adjunctive regimen may help address two competing goals during induction i.e., preserving cardiovascular stability while attenuating airway and sympathetic responses.

Heart rate and mean arterial pressure are routinely used to assess hemodynamic responses during induction and airway instrumentation. However, these variables mainly describe systemic cardiovascular responses and may not fully reflect changes in peripheral vascular tone or peripheral perfusion. This distinction is important because restoration or preservation of MAP does not necessarily imply preservation of peripheral perfusion, particularly when vascular tone is changing rapidly, or vasoactive drugs are administered. During induction, sympathetic withdrawal, propofol-related vasodilation, opioid-related sympatholysis, laryngoscopy-induced sympathetic activation, and vasopressor treatment may all alter peripheral perfusion before, or without, proportional changes in conventional hemodynamic variables. Perfusion index, derived from pulse oximetry, is defined as the ratio of the pulsatile to non-pulsatile component of the photoplethysmographic signal and provides a continuous, noninvasive estimate of peripheral perfusion [21]. Because perfusion index reflects interactions between peripheral vascular tone, stroke volume, and central hemodynamic status, it may provide additional information beyond heart rate and mean arterial pressure during the rapidly changing autonomic conditions of anesthetic induction and intubation [22]. Its use in this setting may therefore strengthen hemodynamic assessment by adding a peripheral perfusion dimension to conventional monitoring. However, evidence regarding its utility during induction of general anesthesia remains limited [23].

Therefore, this pilot study was designed to evaluate whether adding low-dose ketamine and intravenous lidocaine to a propofol-based induction regimen could attenuate early peri-induction hemodynamic disturbance and improve peripheral perfusion compared with a standard propofol–fentanyl regimen. The rationale was preventive rather than rescue-based i.e., instead of relying only on correction of hypotension after it occurs, the adjunctive regimen was intended to explore whether a more physiologically balanced combination of induction agents could reduce the magnitude of MAP reduction, support peripheral perfusion, and attenuate airway-related responses during induction and endotracheal intubation. We used perfusion index together with heart rate and mean arterial pressure as complementary monitoring parameters. We hypothesized that the ketamine–lidocaine adjunctive regimen would be associated with greater MAP stability and higher perfusion index values during induction and intubation compared with the standard propofol–fentanyl regimen.

## 2. Materials and Methods

The study was approved by the Ethics Committee of Riga Stradiņš University, Riga, Latvia (Approval No. 2-PĒK-4/282/2026), and was conducted in accordance with the Declaration of Helsinki. Written informed consent was obtained from all participants before enrolment. The study was conducted as an observational investigation to evaluate perfusion index and hemodynamic responses during routine anesthetic induction.

This was a prospective non—blinded randomized pilot study at Pauls Stradiņš Clinical University Hospital, Riga, Latvia. Consecutive adult patients aged 18 years or older scheduled for elective surgery requiring general anesthesia with endotracheal intubation were screened for inclusion. Patients were randomly assigned to one of the two induction-regimen groups in a 1:1 ratio using a computer-generated randomization sequence prepared before patient enrolment. Allocation was concealed in sequentially numbered, opaque, sealed envelopes, which were opened only after patient inclusion and immediately before induction of anesthesia. Each participant therefore had an equal probability of allocation to either the standard propofol–fentanyl regimen or the ketamine–lidocaine adjunctive regimen.

Patients were excluded if tracheal intubation required more than one attempt, if they had contraindications to any of the induction agents used in the study, American Society of Anesthesiologists physical status III–IV, peripheral vascular disease, Raynaud’s disease, pregnancy, or refusal to participate. A total of 30 patients were enrolled, with 15 patients included in each induction-regimen group.

Patients were included as per their randomization in one of two groups according to the anesthetic induction regimen used. In the standard induction group (Group 1), patients received propofol 2 mg/kg, fentanyl 2 μg/kg, and a neuromuscular blocking agent. In the ketamine–lidocaine adjunctive group (Group 2), patients received propofol 2 mg/kg, ketamine 0.4 mg/kg, lidocaine 1 mg/kg intravenously, fentanyl 1 μg/kg intravenously, and a neuromuscular blocking agent. The fentanyl dose was lower in the ketamine–lidocaine adjunctive group because ketamine and lidocaine were used as additional analgesic and airway-reflex-modulating agents, allowing partial reduction of opioid dose within the induction regimen. Following induction of anesthesia and administration of neuromuscular blockade, endotracheal intubation was performed according to standard institutional practice.

Standard intraoperative monitoring was applied to all patients, including heart rate, non-invasive blood pressure, peripheral oxygen saturation, and perfusion index. Hemodynamic and perfusion index measurements were recorded at three predefined time points: before administration of induction agents, after completion of anesthetic induction and before laryngoscopy, and after endotracheal intubation following confirmation of tracheal tube placement. Perfusion index was obtained from the pulse oximetry signal and recorded when the waveform and signal quality were considered stable. The perfusion index was measured using the pulse oximetry sensor integrated into the standard Philips IntelliVue monitor. The pulse oximeter probe was placed on the patient’s index finger and maintained at the same site throughout the study period.

Hemodynamic variables and perfusion index (PI) were recorded at predefined time points throughout the induction–intubation period. Baseline measurements were obtained before the administration of induction agents. Following completion of induction drug administration, measurements were recorded at 30 s, 1 min, and 3 min. After successful endotracheal intubation, additional measurements were obtained at 30 s, 1 min, and 3 min. At each post-induction and post-intubation time point, PI values were recorded only after a stable pulse oximetry waveform and adequate signal quality had been achieved, and the average of three consecutive measurements was used for analysis. The need for corrective hemodynamic interventions during the induction–intubation period, including vasopressor administration or intravenous fluid bolus for clinically significant hypotension, was also documented.

Perioperative management was standardized for both study groups according to the institutional anaesthesia protocol. Patients followed the same preoperative fasting recommendations. No routine intravenous fluid preload was administered before induction. Intravenous crystalloid administration during the induction period and any subsequent hemodynamic interventions were performed according to the same institutional protocol in both groups.

Cough reflex during intubation was recorded as present or absent based on visible coughing, bucking, or airway reaction during laryngoscopy, tracheal tube passage, or immediately after intubation. Postoperative sore throat severity was assessed using a 10-point numerical rating scale, where 0 indicated no sore throat and 10 indicated the worst imaginable sore throat. Postoperative sore throat severity was assessed approximately 30 min after extubation, once the patient was fully awake and able to provide a reliable self-assessment.

### Statistical Analysis

Continuous variables are presented as mean ± standard deviation and median because of the small pilot sample size and the possibility of non-normal distribution for some physiological variables. Mean ± standard deviation was retained to allow comparison with previous anesthesia studies and to correspond with the reported parametric comparisons, while median values were included to provide an additional measure of central tendency less influenced by extreme observations. Normality of continuous variables was assessed using the Shapiro–Wilk test.

Between-group comparisons were performed using an independent-samples *t*-test or Mann–Whitney U test according to data distribution. Within-group changes in MAP across measurement time points were assessed using paired-samples *t*-tests or non-parametric alternatives where appropriate. Categorical variables were compared using chi-square or Fisher’s exact test, as appropriate. A two-sided *p*-value < 0.05 was considered statistically significant.

## 3. Results

A total of 30 patients were enrolled, with 15 patients included in each induction-regimen group. Baseline demographic and clinical characteristics were comparable between the groups, with no statistically significant differences in ASA physical status, age, height, body weight, or ideal body weight (Table 1).

Baseline hemodynamic parameters were broadly comparable between the two groups (Table 2).

**Figure 1:**
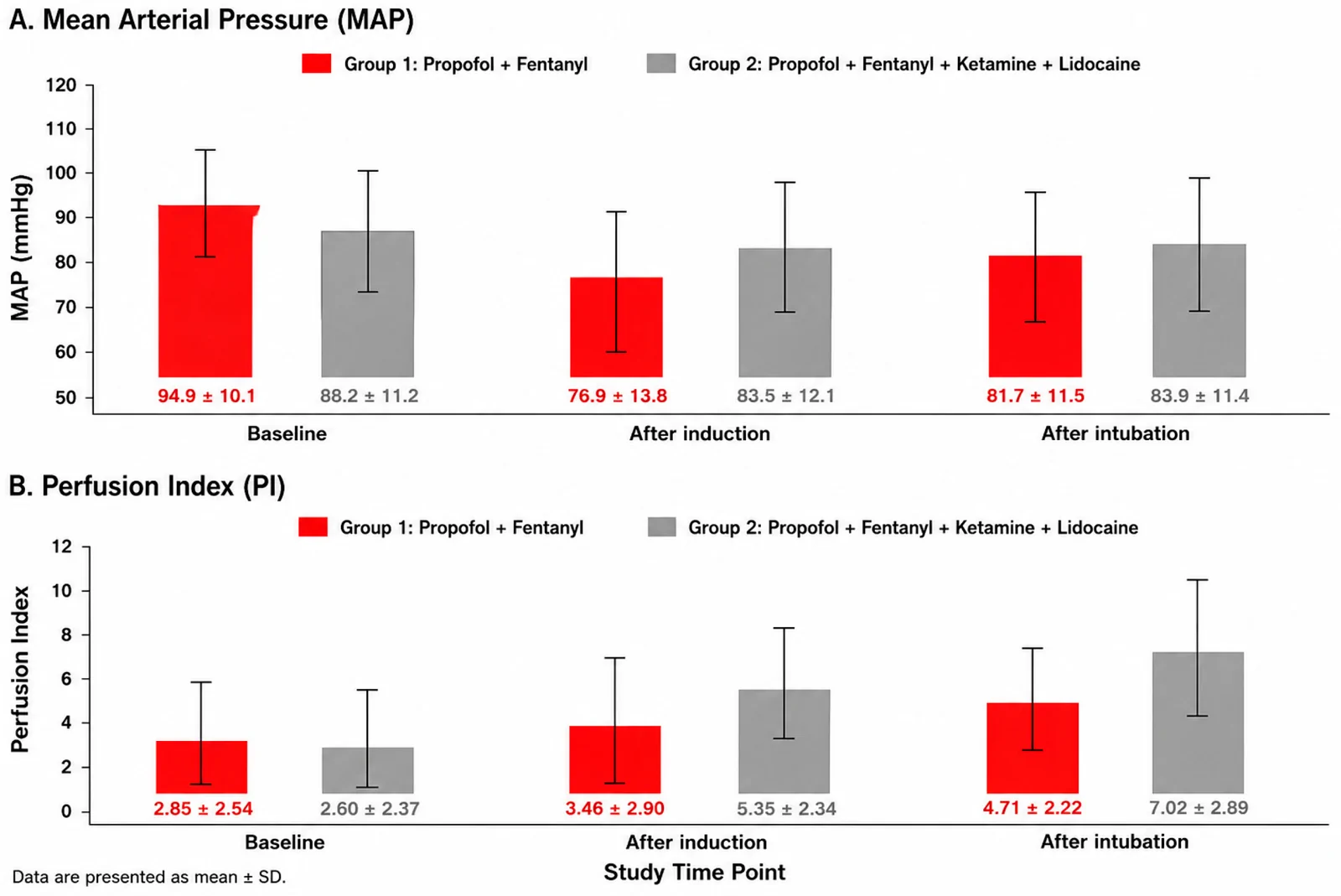
Changes in Mean Arterial Pressure and Perfusion Index Following Anesthetic Induction and Endotracheal Intubation. Changes in mean arterial pressure and perfusion index across peri-induction time points. Bar charts showing changes in mean arterial pressure and perfusion index at baseline, after induction, and after endotracheal intubation in both groups. Data are presented as mean ± SD. Panel (**A**) shows mean arterial pressure, and Panel (**B**) shows perfusion index. Group 1 received propofol, fentanyl, and a neuromuscular blocking agent, while Group 2 received propofol, fentanyl, ketamine, lidocaine, and a neuromuscular blocking agent. The central line represents the median, boxes represent the interquartile range, and whiskers represent the data range. Values displayed below the perfusion index boxplots are mean ± standard deviation. MAP = mean arterial pressure.

No statistically significant between-group differences were observed in heart rate, systolic blood pressure, diastolic blood pressure, mean arterial pressure (MAP), or peripheral oxygen saturation at baseline, after induction, or after endotracheal intubation. No patient in either group required corrective hemodynamic treatment with vasopressors or intravenous fluid bolus for clinically significant hypotension during the induction–intubation observation period. Following induction, MAP decreased from 94.9 ± 10.1 mmHg to 76.9 ± 13.8 mmHg in Group 1, corresponding to a mean reduction of 18 mmHg. In Group 2, MAP decreased from 88.2 ± 11.2 mmHg to 83.5 ± 12.1 mmHg, corresponding to a smaller mean reduction of 4.7 mmHg. After intubation, MAP was 81.7 ± 11.5 mmHg in Group 1 and 83.9 ± 11.4 mmHg in Group 2, corresponding to reductions from baseline of 13.2 mmHg and 4.3 mmHg, respectively. Between-group MAP differences were not statistically significant after induction or after intubation (Table 2; Figure 1).

Within-group MAP changes are presented in Table 3. In Group 1, MAP decreased significantly after induction compared with baseline (*p* < 0.001) and remained significantly lower than baseline after intubation (*p* = 0.001). No significant difference was observed between post-induction and post-intubation MAP values in Group 1 (*p* = 0.133). In Group 2, MAP showed a smaller decrease after induction and partial recovery after intubation, but none of the within-group MAP changes reached statistical significance. These findings suggest improved overall hemodynamic stability in the ketamine–lidocaine adjunctive group, although no statistically significant between-group differences in mean arterial pressure (MAP) were observed at individual time points (Figure 1).

Perfusion index values are shown in Table 4 and Figure 1. Baseline perfusion index was similar between the groups, with mean values of 2.85 ± 2.54 in Group 1 and 2.60 ± 2.37 in Group 2. After induction, perfusion index increased to 3.46 ± 2.90 in Group 1 and 5.35 ± 2.34 in Group 2, corresponding to mean increases from baseline of 0.61 and 2.75, respectively. This between-group difference was statistically significant (*p* = 0.043). After intubation, perfusion index increased further to 4.71 ± 2.22 in Group 1 and 7.02 ± 2.89 in Group 2, corresponding to mean increases from baseline of 1.86 and 4.42, respectively. The between-group difference remained statistically significant after intubation (*p* = 0.020). These findings suggest that the ketamine–lidocaine adjunctive regimen was associated with higher peripheral perfusion index values during induction and intubation compared with the standard propofol–fentanyl regimen.

Postoperative sore throat NRS scores were low in both groups but were significantly lower in Group 2 than in Group 1, with mean values of 0.53 ± 0.91 and 1.67 ± 1.76, respectively (*p* = 0.035). Given the small sample size, this airway-related outcome should be interpreted as exploratory. The incidence of cough following endotracheal intubation was also significantly lower in Group 2 than in Group 1. Cough was observed in 3 of 15 patients (20.0%) in Group 2 compared with 13 of 15 patients (86.7%) in Group 1 (Fisher’s exact test, *p* < 0.001). These findings suggest greater suppression of airway reflexes in the ketamine–lidocaine adjunctive group. However, as this was a secondary outcome assessed in a small randomized cohort, the results should be considered exploratory and require confirmation in larger prospective studies.

## 4. Discussion

The main finding of this pilot study was that the addition of low-dose ketamine and intravenous lidocaine to propofol-based induction was associated with a more stable within-group MAP pattern and significantly higher perfusion index values after induction and after endotracheal intubation compared with standard propofol–fentanyl induction. Although between-group MAP differences at individual time points were not statistically significant, MAP decreased significantly from baseline in the propofol–fentanyl group and remained lower after intubation, whereas no statistically significant within-group MAP reduction was observed in the ketamine–lidocaine group. These findings should therefore be interpreted as an exploratory physiological signal rather than as definitive evidence of superior hemodynamic stability.

The decrease in MAP observed in the propofol–fentanyl group is consistent with the known cardiovascular effects of propofol, including systemic vasodilation, reduced sympathetic tone, and myocardial depression, particularly when combined with opioids [24,25]. In contrast, the smaller MAP reduction observed in the ketamine–lidocaine group may reflect the complementary pharmacological effects of the adjunctive agents. Ketamine may partially counterbalance propofol-induced hypotension through sympathetic stimulation, preservation of cardiac output, and maintenance of vascular tone [26,27]. Lidocaine may additionally attenuate airway reflexes and reduce the hemodynamic response associated with laryngoscopy and endotracheal intubation [28]. These mechanisms provide a biologically plausible explanation for the observed pattern of greater hemodynamic stability in the adjunctive-regimen group, although no statistically significant between-group differences in mean arterial pressure (MAP) were detected.

A central interpretative issue is that the hemodynamic rationale for evaluating an adjunctive induction regimen was not clinically confirmed in this small pilot cohort. Although propofol–opioid induction is known to be associated with hypotension in previous literature, and may require corrective treatment in susceptible patients, none of the patients in the standard propofol–fentanyl group in the present study required vasopressor administration or intravenous fluid bolus for clinically significant hypotension. Therefore, the present data do not show that the ketamine–lidocaine regimen prevented correction-requiring hypotension compared with standard induction. The observed hemodynamic difference was limited to the pattern of MAP change: MAP decreased significantly from baseline in the propofol–fentanyl group, whereas the reduction was smaller and statistically non-significant in the ketamine–lidocaine group. This finding should be interpreted as a physiological signal rather than a clinically proven advantage. Consequently, the justification for further evaluation of this regimen cannot rest on hemodynamic rescue-treatment reduction alone but should be based on the combined findings of MAP pattern, higher perfusion index values, and reduced airway-related responses, together with the existing literature suggesting that propofol–opioid induction may produce clinically relevant hypotension in larger or higher-risk populations.

Perfusion index increased in both groups after induction and intubation, but values were significantly higher in the ketamine–lidocaine group at both post-induction and post-intubation time points. PI is influenced by several physiological factors, including peripheral vascular tone, pulsatile blood flow, stroke volume, and sympathetic activity [21,29]. Therefore, the higher PI values observed in Group 2 may reflect reduced peripheral vascular tone and higher peripheral perfusion index during induction and intubation. However, PI should be interpreted as an indirect marker of peripheral perfusion rather than as definitive evidence of improved microcirculatory function or clinical outcome [30]. Importantly, the higher PI values in Group 2 occurred without a statistically significant fall in MAP, suggesting that the adjunctive regimen may have supported peripheral perfusion while maintaining systemic hemodynamic stability. This finding is consistent with previous work suggesting that S-ketamine administration during anesthesia induction may increase PI while preserving arterial pressure [30]. The higher PI observed in the ketamine–lidocaine group should be interpreted with caution. Although PI is commonly used as a non-invasive surrogate of peripheral perfusion, it is strongly influenced by peripheral vascular tone, sympathetic activity, intravascular volume status, and temperature. Therefore, increased PI values do not necessarily indicate superior tissue perfusion or a clinically improved hemodynamic state.

The clinical relevance of PI in this context is not that it should replace conventional hemodynamic monitoring, but that it may provide complementary information during the short and physiologically unstable transition from awake sympathetic tone to anesthetized vasodilated physiology. MAP and heart rate reflect systemic hemodynamic status, but they may not fully capture changes in peripheral vascular tone or redistribution of blood flow during induction. In the present study, PI appeared to distinguish the two induction-regimen patterns more clearly than MAP alone. This supports the potential value of PI as an additional non-invasive monitoring parameter during anesthesia induction, particularly in studies evaluating induction-related vascular and perfusion responses.

Postoperative sore throat scores were also lower in the ketamine–lidocaine group. This finding is biologically plausible, particularly because intravenous lidocaine has been associated with attenuation of airway reflexes and reduction of postoperative airway discomfort [31]. Cough-reflex scores were also lower in the ketamine–lidocaine group, suggesting greater suppression of airway reflexes during intubation. However, both airway-related outcomes should be interpreted cautiously because the sample size was small, and these outcomes were exploratory.

This study has several limitations. First, the sample size was small, with only 15 patients in each group, limiting statistical power and making the findings exploratory rather than confirmatory. Second, randomized allocation reduces selection bias; however, because this was a small pilot study with only 15 patients per group, randomization may not have fully eliminated baseline imbalances or residual confounding. Third, the study included relatively low-risk elective surgical patients, which limits generalizability to older, frailer, or hemodynamically unstable populations. Fourth, perfusion index is influenced by multiple factors, including peripheral vascular tone, temperature, sensor position, and local perfusion, and should therefore be interpreted alongside conventional hemodynamic variables [21,32]. Fifth, the combined use of ketamine and lidocaine means that the individual contribution of each adjunctive agent cannot be separated. The difference in fentanyl dose between groups should also be considered when interpreting the findings. Although the lower fentanyl dose in the ketamine–lidocaine group reflects the intended opioid-sparing nature of the adjunctive regimen, it also means that the observed hemodynamic and perfusion-index patterns cannot be attributed solely to ketamine and lidocaine.

Finally, the study was not powered to assess clinical outcomes such as postoperative complications, recovery quality, vasopressor requirement, or adverse airway events. In addition, because no patient in either group required corrective vasopressor therapy or intravenous fluid bolus for hypotension, the present study cannot determine whether the adjunctive regimen reduces the need for rescue hemodynamic treatment.

This was a prospective randomized, non-blinded pilot study. Although randomization was performed, blinding of participants and investigators was not feasible due to the nature of the intervention. Consequently, assessment of subjective outcomes, such as postoperative cough and sore throat, may have been influenced by observer bias and should therefore be interpreted with caution.

Future randomized studies should evaluate whether PI changes during induction are reproducible in larger and more heterogeneous patient populations, including older patients and patients with limited cardiovascular reserve. Further studies should also examine whether PI-guided assessment during induction is associated with clinically meaningful outcomes such as hypotension requiring vasopressor therapy, airway reflex suppression, postoperative sore throat, recovery quality, or perioperative adverse events. Such studies may help clarify whether PI is simply a sensitive physiological marker or whether it has practical value in guiding induction-regimen selection. Furthermore, PI is an indirect physiological marker and should not be interpreted as a direct measure of tissue perfusion or microcirculatory function. Additional methods of perfusion assessment would be required to confirm whether the observed differences represent clinically meaningful improvements in peripheral or systemic perfusion.

## 5. Conclusions

The addition of low-dose ketamine and intravenous lidocaine to propofol-based induction was associated with a more stable within-group MAP pattern and significantly higher perfusion index values after induction and endotracheal intubation. Postoperative sore throat scores were also lower in the adjunctive-regimen group. These findings suggest a potential hemodynamic benefit and possible airway-related effect of this regimen, but given the small sample size and observational design, the results should be considered hypothesis-generating and require confirmation in larger randomized studies.

## Figures and Tables

**Table 1:** Baseline characteristics of the study groups.

Variable	Group 1, *n* = 15	Group 2, *n* = 15	*p*-Value
ASA physical status	1.40 ± 0.50 (1.40)	1.47 ± 0.50 (1.40)	0.72
Age, years	51.6 ± 13.1 (49.0)	50.3 ± 18.3 (55.0)	0.81
Height, cm	175.3 ± 8.5 (174.0)	170.8 ± 7.2 (172.0)	0.10
Weight, kg	76.8 ± 7.9 (80.0)	72.8 ± 7.0 (74.0)	0.16
Ideal body weight, kg	70.5 ± 5.9 (70.6)	65.8 ± 8.1 (66.0)	0.09

Data are presented as mean, median, standard deviation (SD). Group 1 = standard induction group; Group 2 = ketamine–lidocaine induction group. ASA = American Society of Anesthesiologists physical status classification. *p*-values compare Group 1 and Group 2; *p* < 0.05 was considered statistically significant.

**Table 2:** Hemodynamic parameters at baseline, after induction, and after intubation.

Time Pont	Variable	Group 1 (*n* = 15)	Group 2 (*n* = 15)	*p*-Value
Baseline	HR	78.8 ± 11.8 (78.0)	87.4 ± 15.0 (87.5)	0.110
	BP systolic	138.3 ± 11.7 (140.0)	128.5 ± 18.2 (124.0)	0.094
	BP diastolic	80.7 ± 9.9 (79.0)	78.3 ± 11.9 (75.0)	0.555
	MAP	94.9 ± 10.1 (92.0)	88.2 ± 11.2 (88.0)	0.100
	SpO_2_	98.5 ± 2.2 (100.0)	99.7 ± 0.7 (100.0)	0.054
After Induction	HR	74.7 ± 11.0 (71.0)	81.7 ± 14.3 (80.0)	0.157
	BP systolic	105.3 ± 26.0 (97.0)	113.0 ± 21.5 (105.0)	0.386
	BP diastolic	66.3 ± 12.7 (65.0)	69.7 ± 13.1 (65.0)	0.478
	MAP	76.9 ± 13.8 (75.0)	83.5 ± 12.1 (80.0)	0.493
	SpO_2_	99.0 ± 1.4 (100.0)	99.7 ± 0.5 (100.0)	0.075
After Intubation	HR	78.9 ± 12.3 (80.0)	87.6 ± 11.8 (80.0)	0.076
	BP systolic	110.1 ± 19.0 (110.0)	113.1 ± 10.0 (115.5)	0.560
	BP diastolic	74.6 ± 14.8 (73.0)	75.5 ± 12.5 (78.0)	0.864
	MAP	81.7 ± 11.5 (88.0)	83.9 ± 11.4 (85.0)	0.605
	SpO_2_	99.7 ± 0.8 (100.0)	99.7 ± 0.5 (100.0)	0.136
Induction- intubation period	Corrective vasopressor/fluid bolus required	0/15 (0%)	0/15 (0%)	

Values are presented as mean ± standard deviation (median) for Group 1 and Group 2. Intergroup comparisons were performed at each time point. A *p*-value < 0.05 was considered statistically significant. HR = heart rate; BP = blood pressure; MAP = mean arterial pressure; SpO_2_ = peripheral oxygen saturation. HR is reported as beats/min, BP and MAP as mmHg, and SpO_2_ as percentage. No statistically significant differences were observed between groups at baseline, after induction, or after intubation. Note: The SpO_2_ after intubation unit has been corrected to %.

**Table 3:** Within-Group Changes in Mean Arterial Pressure.

Group	Comparison	Mean Difference, mmHg	T	df	*p*-Value
Group 1	Baseline vs. after induction	18.0	4.42	14	<0.001
Group 1	Baseline vs. after intubation	13.2	3.97	14	0.001
Group 1	After induction vs. after intubation	−4.8	−1.59	14	0.133
Group 2	Baseline vs. after induction	4.73	1.85	14	0.085
Group 2	Baseline vs. after intubation	4.33	1.82	14	0.090
Group 2	After induction vs. after intubation	−0.400	−0.121	14	0.906

Mean differences are calculated as the first measurement minus the second measurement. Positive values therefore indicate a reduction in MAP from the first to the second timepoint, while negative values indicate an increase. Paired-samples *t*-tests were used for within-group comparisons. Group 1 received propofol, fentanyl, and a neuromuscular blocking agent. Group 2 received propofol, fentanyl, ketamine, lidocaine, and a neuromuscular blocking agent. MAP = mean arterial pressure.

**Table 4:** Perfusion index at individual time points in both groups.

Time Point	Group 1, Mean ± SD	Group 2 Mean ± SD	*p*-Value
Baseline	2.85 ± 2.54	2.60 ± 2.37	0.787
After induction	3.46 ± 2.90	5.35 ± 2.34	0.043
After intubation	4.71 ± 2.22	7.02 ± 2.89	0.020

Values are presented as mean ± standard deviation and median. *N* = 15 in each group. *p*-values indicate between-group comparisons at each time point. Group 1: Propofol–fentanyl, Group 2: Propofol–fentanyl–ketamine–lidocaine.

## Data Availability

The original contributions presented in this study are included in the article. Further inquiries can be directed to the corresponding author.

## Abbreviations

The following abbreviations are used in this manuscript:

ASA	American Society of Anesthesiologists
BP	Blood Pressure
HR	Heart Rate
IBW	Ideal Body Weight
MAP	Mean Arterial Pressure
NMDA	N-Methyl-D-Aspartate
NRS	Numerical Rating Scale
PI	Perfusion Index
SD	Standard Deviation
SpO_2_	Peripheral Oxygen Saturation
